# Reliability of Automated RECIST 1.1 and Volumetric RECIST Target Lesion Response Evaluation in Follow-Up CT—A Multi-Center, Multi-Observer Reading Study

**DOI:** 10.3390/cancers16234009

**Published:** 2024-11-29

**Authors:** Isabel C. Dahm, Manuel Kolb, Sebastian Altmann, Konstantin Nikolaou, Sergios Gatidis, Ahmed E. Othman, Alessa Hering, Jan H. Moltz, Felix Peisen

**Affiliations:** 1 Department of Diagnostic and Interventional Radiology, Eberhard Karls University, Tuebingen University Hospital, Hoppe-Seyler-Str. 3, 72076 Tuebingen, Germany; 2 Department of Radiology, Te Whatu Ora Waikato, Hamilton 3240, New Zealand; 3 Institute of Neuroradiology, Johannes Gutenberg University Hospital Mainz, Langenbeckstr. 1, 55131 Mainz, Germany; 4 Image-Guided and Functionally Instructed Tumor Therapies (iFIT), The Cluster of Excellence (EXC 2180), 72076 Tuebingen, Germany; 5 Fraunhofer MEVIS, Max-von-Laue-Str. 2, 28359 Bremen, Germany; 6 Diagnostic Image Analysis Group, Radboudumc, Geert Grooteplein Zuid 10, 6525 GA Nijmegen, The Netherlands

**Keywords:** melanoma, artificial intelligence and machine learning, RECIST 1.1

## Abstract

Objectives: To evaluate the performance of a custom-made convolutional neural network (CNN) algorithm for fully automated lesion tracking and segmentation, as well as RECIST 1.1 evaluation, in longitudinal computed tomography (CT) studies compared to a manual Response Evaluation Criteria in Solid Tumors (RECIST 1.1) evaluation performed by three radiologists. Methods: Baseline and follow-up CTs of patients with stage IV melanoma (n = 58) was investigated in a retrospective reading study. Three radiologists performed manual measurements of metastatic lesions. Fully automated segmentations were generated, and diameters and volumes were computed from the segmentation results, with subsequent RECIST 1.1 evaluation. We measured (1) the intra- and inter-reader variability in the manual diameter measurements, (2) the agreement between manual and automated diameter measurements, as well as the resulting RECIST 1.1 categories, and (3) the agreement between the RECIST 1.1 categories derived from automated diameter measurement compared to automated volume measurements. Results: In total, 114 target lesions were measured at baseline and follow-up. The intraclass correlation coefficients (ICCs) for the intra- and inter-reader reliability of the diameter measurements were excellent, being >0.90 for all readers. There was moderate to almost perfect agreement when comparing the timepoint response category derived from the mean manual diameter measurements from all three readers with those derived from automated diameter measurements (Cohen’s k 0.67–0.76). The agreement between the manual and automated volumetric timepoint responses was substantial (Fleiss’ k 0.66–0.68) and that between the automated diameter and volume timepoint responses was substantial to almost perfect (Cohen’s k 0.81). Conclusions: The automated diameter measurement of preselected target lesions in follow-up CT is reliable and can potentially help to accelerate RECIST evaluation.

## Simple Summary

The Response Evaluation Criteria in Solid Tumors (RECIST) 1.1 are the current standard in assessing tumor dynamics in cross-sectional imaging. Although they provide standardized criteria, several sources of variability remain, such as the susceptibility of manual measurements to interobserver variability, especially when defining the tumor margins in irregularly shaped lesions, or the limitations of diameter-based measurements when assessing changes in the sizes of non-spherical tumors. Algorithms for automated segmentation may offer an opportunity to reduce this variability, provided that they can be implemented into the routine workflow without compromising the reading time. We evaluated a convolutional neural network (CNN)-based algorithm for fully automated lesion tracking and segmentation in longitudinal CT studies and subsequent RECIST 1.1 evaluation and confirmed that its automated diameter and volume measurements of preselected target lesions are reliable and can accelerate RECIST evaluations.

## 1. Introduction

According to the current standards, the tumor response to systemic oncological treatment is monitored by cross-sectional imaging, requiring standardized models for the evaluation of changes in the sizes of tumor lesions to assess the overall treatment response. With this aim, the Response Evaluation Criteria in Solid Tumors (RECIST) were originally introduced in the late 1990s and later revised in 2009 (RECIST 1.1), proposing a unidimensional measurement model to estimate the overall tumor burden [1,2].

At baseline, lesions are selected as either target lesions (TL) or non-target lesions (NTL). At each subsequent timepoint, these lesions are measured or evaluated to define one of four categories of response: complete response (CR), partial response (PR), stable disease (SD), and progressive disease (PD).

Despite the presence of standardized criteria for objective assessment, RECIST 1.1 exhibits several sources of variability, including intra- and interobserver variability associated with manual measurements and target lesion selection, as well as limitations to diameter-based measurements when assessing non-spherical lesions [3,4,5]. Multiple studies have shown that the response evaluation derived from volumetric tumor measurements differs significantly from that derived from uni- or bidimensional measurements, due to the better representation of irregularly shaped lesions [6]. However, in most cases, the particular software for semi-automated segmentation was evaluated without considering the clinical routine workflow, leading to compromises in the overall reading time [7].

Recent advances in imaging analysis using neural networks have resulted in several algorithms that enable the reliable automation of lesion tracking and segmentation, with a subsequent reduction in intra- and inter-reader variability and the potential shortening of the reading time. However, many algorithms focus on single lesion types, such as liver metastases [8,9]. Universal lesion segmentation algorithms offer the advantage of not requiring manual lesion type selection and often generalize better to further lesion types not seen in training [10,11,12]. Nonetheless, such algorithms remain scarce and are rarely tested against multiple human readers to assess their reliability.

Based on the manual segmentation of over 16,000 lesions, covering a broad range of lesion types (lymphatic metastases, parenchymatous organ metastases, osseous metastases, and soft tissue metastases), we developed a convolutional neural network (CNN)-based algorithm for fully automated lesion tracking and segmentation in longitudinal computed tomography (CT) studies and subsequent RECIST 1.1 evaluation. In tumor segmentation, CNNs address several key challenges. Tumors often exhibit irregular shapes, varying sizes, and complex textures, making manual segmentation both time-consuming and error-prone. CNNs, with hierarchical feature learning capabilities, automatically extract important features at multiple scales, helping to detect even subtle tumor boundaries accurately. Another significant advantage of CNNs in tumor segmentation is their ability to learn from annotated data, allowing them to generalize well to new, unseen patient scans after adequate training [13]. In the present study, this algorithm was tested against three radiologists from three different institutions in a dataset of 58 patients with metastatic melanoma.

The purpose of the study was (1) to evaluate the intra- and inter-reader variability in manual diameter measurements of TLs and the corresponding timepoint responses in longitudinal CT studies, (2) to assess the agreement between manual and automated diameter measurements, as well as their resulting timepoint responses, and (3) to investigate the agreement between timepoint responses resulting from manual and automated diameter measurements and those resulting from automated volumetric measurements.

## 2. Materials and Methods

### 2.1. Sample

The testing sample comprised 58 patients, randomly selected from the local melanoma registry, who received their baseline CT between 2015 and 2018. These patients were excluded from the training cohort used for the proposed automated registration and segmentation algorithm.

### 2.2. Imaging

A total of 47 baseline and 52 follow-up CT scans were acquired in the local department of radiology on five different scanners. An additional 11 baseline and 6 follow-up CT scans were acquired at external locations. Complete information regarding the scanning protocols for these scans is unavailable. All CT scans were obtained in the portal venous phase. For a detailed analysis of the distribution of the CT scanners and detailed information about the scanning protocols of in-house CT scanners, refer to Table A1 in Appendix A.

### 2.3. Definition of Target Lesions and RECIST Timepoint Response Evaluation

Prior to manual or automated measurement, target lesions were defined on the baseline scans for every patient according to the RECIST 1.1 criteria [1], designated by FP. A marker was displayed in the center of each target lesion, without indicating the lesion’s size. The readers were required to adhere to this selection and could not independently change the lesions or choose new lesions to ensure reproducibility. The subsequent RECIST 1.1 timepoint response evaluation after the first follow-up imaging was based on the predefined set of target lesions. Here, the lesions had to be re-identified by the readers. The appearance of new lesions was not factored into the assessment. 

Additionally, the volumetric RECIST criteria were evaluated. RECIST assumes that tumors are spherical, with proportional changes in the tumor volume and diameter. By extrapolation, the established diametric RECIST thresholds of a 30% decrease for partial response and 20% increase for progressive disease correspond to volumetric thresholds of a 65% decrease and 73% increase, respectively, using the following formula: $V=\frac{3}{4}\pi r^{3}$ [14]. However, several authors argue that empirically determined volumetric thresholds differ from those extrapolated, possibly because the size changes of the largest diameter overestimate the actual size changes in non-spherical lesions [15].

### 2.4. Manual Evaluation

Three radiologists with varying degrees of experience and from three different institutions acted as readers (I.D., University Hospital Tuebingen, Germany, two years of experience in oncological imaging; M.K., Department of Radiology, Hamilton, New Zealand, nine years of experience in oncological imaging; and S.A., University Hospital Mainz, Germany, seven years of experience in oncological imaging). The lesion evaluation was carried out on custom-made segmentation software (SATORI, MEVIS Fraunhofer, Bremen, Germany). The baseline and follow-up images were presented side by side and slice synchronization based on image registration could optionally be activated. The readers manually drew diameters for the predefined target lesions in the baseline and first follow-up CT, according to the RECIST 1.1 criteria (longest possible diameter on axial slice for non-lymph node lesions, short axis for lymph nodes). Before the segmentation of the test cohort, all readers used a set of training cases to familiarize them with the software. All readers were blinded to the segmentations of the other readers. The manual evaluation was conducted twice per lesion by each reader, with a one-week interval to assess the intra- and inter-reader reliability and minimize recall bias.

### 2.5. Automated Diameter Plotting and Volumetric Segmentation

**Figure 1:**
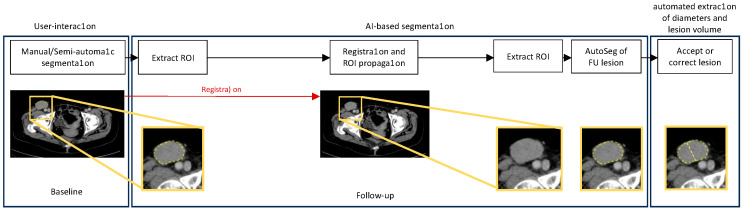
Schema of the proposed pipeline for the AI-assisted segmentation of metastases in follow-up computed tomography (CT) scans. The AI-assisted segmentation pipeline includes four major components: (1) extraction of the region of interest (ROI) around the lesion in the baseline scan; (2) registration of the baseline to the follow-up scan; (3) propagation of the ROI to the follow-up scan to constrain the search region and inference of the trained U-Net to segment all the lesions in the defined region; (4) selection of the corresponding lesion from the output of the nnU-Net.

For automatic measurements, a previously published algorithm was employed [16]. At baseline, the algorithm requires a point within the lesion as input, computed as the center of gravity of FP’s segmentation. The algorithm primarily utilizes an nnUNet [17] that was trained on more than 16,000 lesions from a large variety of CT scans from different hospitals and patients with different primary tumors. NnU-net is a deep learning-based segmentation method that automatically configures itself for the entire segmentation pipeline, including preprocessing, network architecture, training, and the post-processing of biomedical tasks. The training dataset included melanoma cases from University Hospital Tuebingen. For follow-up, the algorithm uses deformable whole-body image registration to re-identify and segment lesions using the nnUNet model (see Figure 1). The nnUNet is capable of omitting segmentations for lesions that may have disappeared due to therapy. The segmentations were generated fully automatically, without visual verification. RECIST diameters (long or short axis, depending on lesion type) and volumes were computed from the segmentation results.

### 2.6. Statistical Analysis

The characterization of the cohort and subsequent statistical tests of the intra- and inter-reader reliability were conducted using IBM SPSS Statistics version 26. The intra-reader reliability of the diameter measurements for individual target lesions (lesion level) and the sum of all target lesions per patient (patient level) were evaluated with intraclass correlation coefficients (ICC), applying a two-way mixed-effects model based on a mean rating (k = 2) and the absolute agreement definition [18,19]. The inter-reader reliability of the target lesion measurements at the lesion and patient level was evaluated with intraclass correlation coefficients, with a two-way random-effects model, based on a mean rating (k = 3) and the absolute agreement definition selected [18,20]. Per definition, an ICC < 0.5 indicates poor reliability, an ICC between 0.5 and 0.75 indicates moderate reliability, an ICC between 0.75 and 0.9 indicates good reliability, and an ICC > 0.90 indicates excellent reliability [18]. The timepoint response agreement between the three readers was evaluated at the lesion and patient level using Fleiss’ Kappa (categorized as poor (k = 0), slight (k = 0–0.20), fair (k = 0.21–0.40), moderate (k = 0.41–0.60), substantial (k = 0.61–0.80), and almost perfect (k > 0.80)) [18,21].

The automated diameter RECIST evaluation was tested against the mean diameters and subsequent timepoint responses of three readers, as well as the mean diameter of all manual measurements, using ICCs (with a two-way random-effects model, based on a mean rating (k = 4) and the absolute agreement definition selected), Fleiss’ Kappa, and Cohen’s Kappa [18].

The comparison of the (automated) diameter RECIST and automated volumetric RECIST was performed with Fleiss’ Kappa and Cohen’s Kappa [18,21].

## 3. Results

### 3.1. Patient Characteristics

Our testing sample consisted of the baseline and first follow-up CT scans of 58 stage IV melanoma patients (AJCC 8th Edition), who received either immunotherapy (69%) or targeted therapy (31%). The cohort included more male than female patients. (Male *n* = 57%), with a mean age of 62.8 years. A median of two target lesions was assigned per patient at baseline, resulting in a total of 114 target lesions measured at both baseline and follow-up. For a detailed description of the testing sample, see Table 1.

### 3.2. Intra-Reader Reliability

The mean differences in the diameter measurements across all readers were minimal, with the individual lesion measurements (lesion level: <1 mm) and the sum of all target lesions per patient (patient level: <2 mm) showing only slight variation. However, the follow-up measurements of one reader (SA) differed significantly between the first and second reading session at both the lesion and patient level (see Table 2). The ICCs for the intra-reader reliability of the diameter measurements at the lesion and patient level were excellent, being > 0.90 for all readers (Table 3).

### 3.3. Inter-Reader Reliability

**Figure 2:**
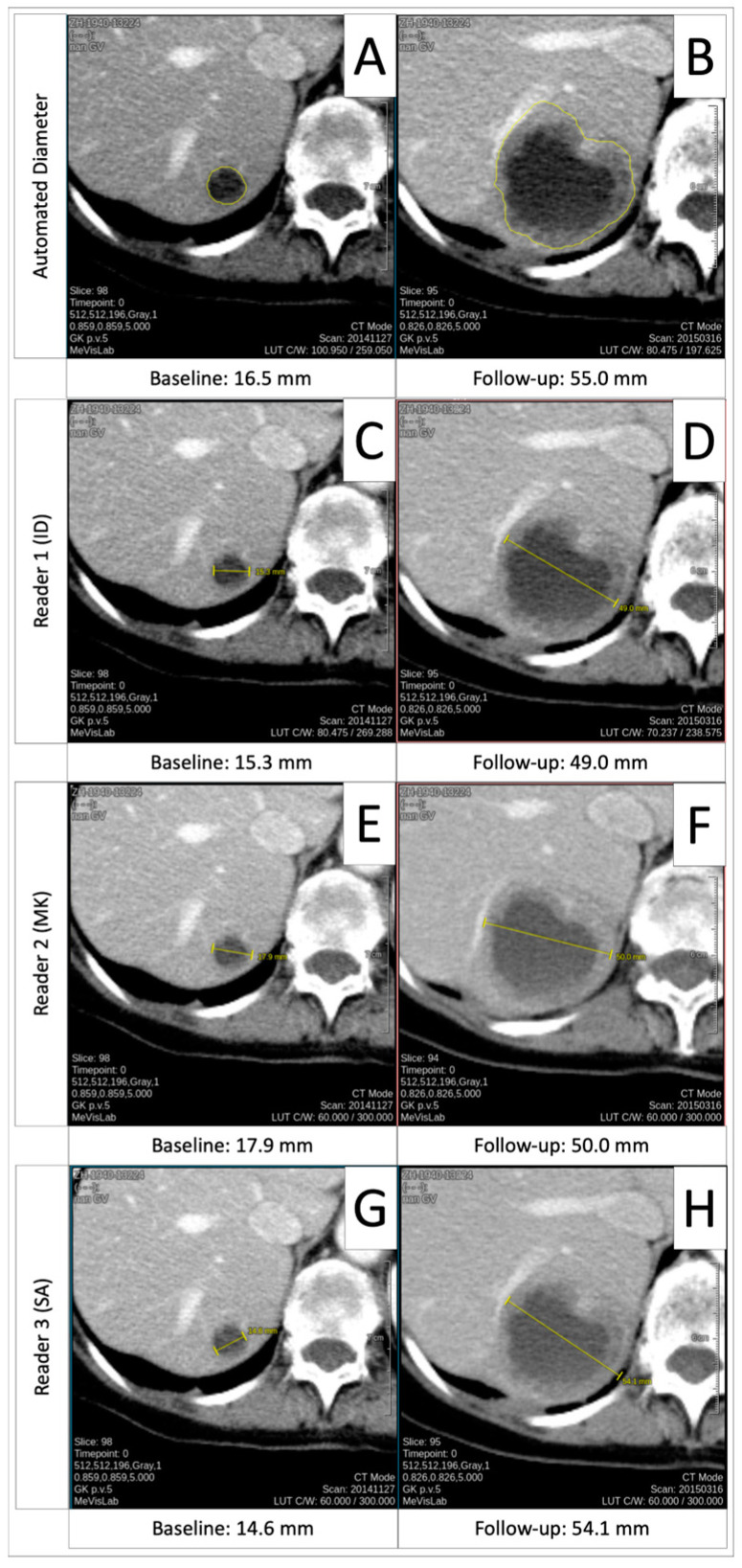
Excellent inter-reader agreement. Exemplary baseline and follow-up measurements for automated diameter (**A**,**B**), reader 1 (**C**,**D**), reader 2 (**E**,**F**), and reader 3 (**G**,**H**), illustrating the excellent inter-reader agreement.

The ICCs for the inter-reader reliability of the diameter measurements among human readers were excellent for both timepoints at the lesion and patient levels, with all timepoints showing ICC values exceeding 0.90 (see Figure 2). The agreement for the timepoint response was substantial at the lesion and patient level (Fleiss’ k 0.68–0.79, Table 4).

### 3.4. Comparison of Automated Diameters to Manual Measurements

The difference between the mean diameters of each reader and the automated diameters was ≤2 mm at the lesion level and ≤4 mm at the patient level. Significant differences were noted for reader M.K. at the baseline at both the lesion and patient levels and for reader S.A. at follow-up at the lesion level. The difference between the calculated mean diameter of all readers and the automated diameters was ≤1 mm at the lesion level and ≤2 mm at the patient level (Table 5).

When incorporating automated diameter measurements, the ICCs remained excellent (>0.90) for both timepoints at the lesion and patient level (Table 4 and Figure 2). Fleiss’ Kappa for the timepoint response continued to indicate substantial agreement at both the lesion and patient levels. When aggregating all readers’ diameters into a single mean manual diameter for timepoint response calculation and comparing it with the automated timepoint response, Cohen’s Kappa indicated moderate to almost perfect agreement (Table 4).

### 3.5. Comparison of Manual and Automated Diameter Timepoint Response to Volumetric Timepoint Response

The agreement between the three readers and the automated volumetric timepoint response was substantial at both the lesion and patient level (Fleiss’ k 0.66–0.68). When all readers’ diameters were aggregated into a single mean manual diameter for timepoint response calculation and compared to the automated volume timepoint response, Cohen’s Kappa ranged from moderate to almost perfect (0.58–0.87). The agreement between the automated diameter and volume timepoint response was substantial to almost perfect (Table 4).

### 3.6. Progressive Disease Timepoint Response Deviation

**Figure A1:**
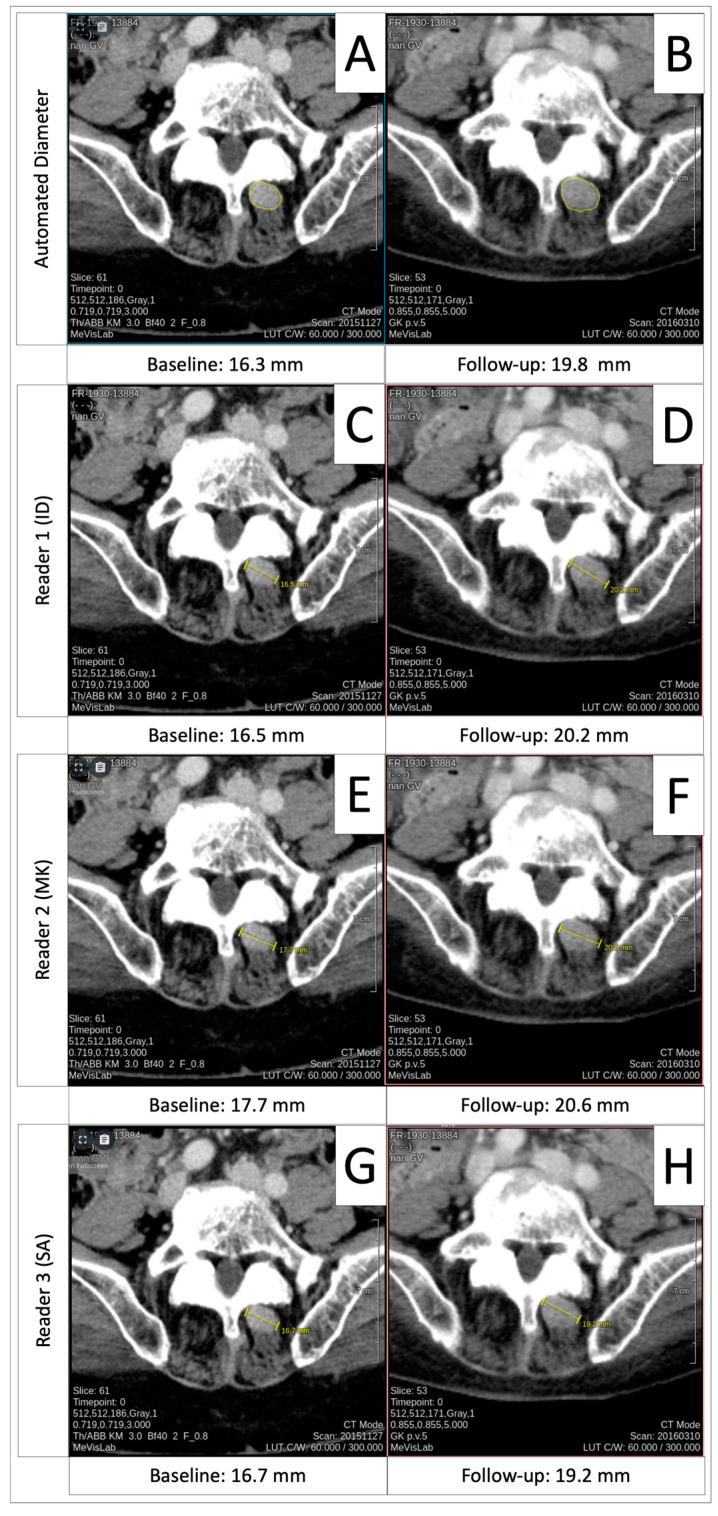
Tumor growth close to 20%, depending on measurement. Baseline and follow-up measurements for automated diameter (**A**,**B**), reader 1 (**C**,**D**), reader 2 (**E**,**F**) and reader 3 (**G**,**H**), with tumor growth close under 20% (automated diameter, reader 2 and 3) or over 20% (reader 1), and resulting differences for timepoint response.

**Figure A3:**
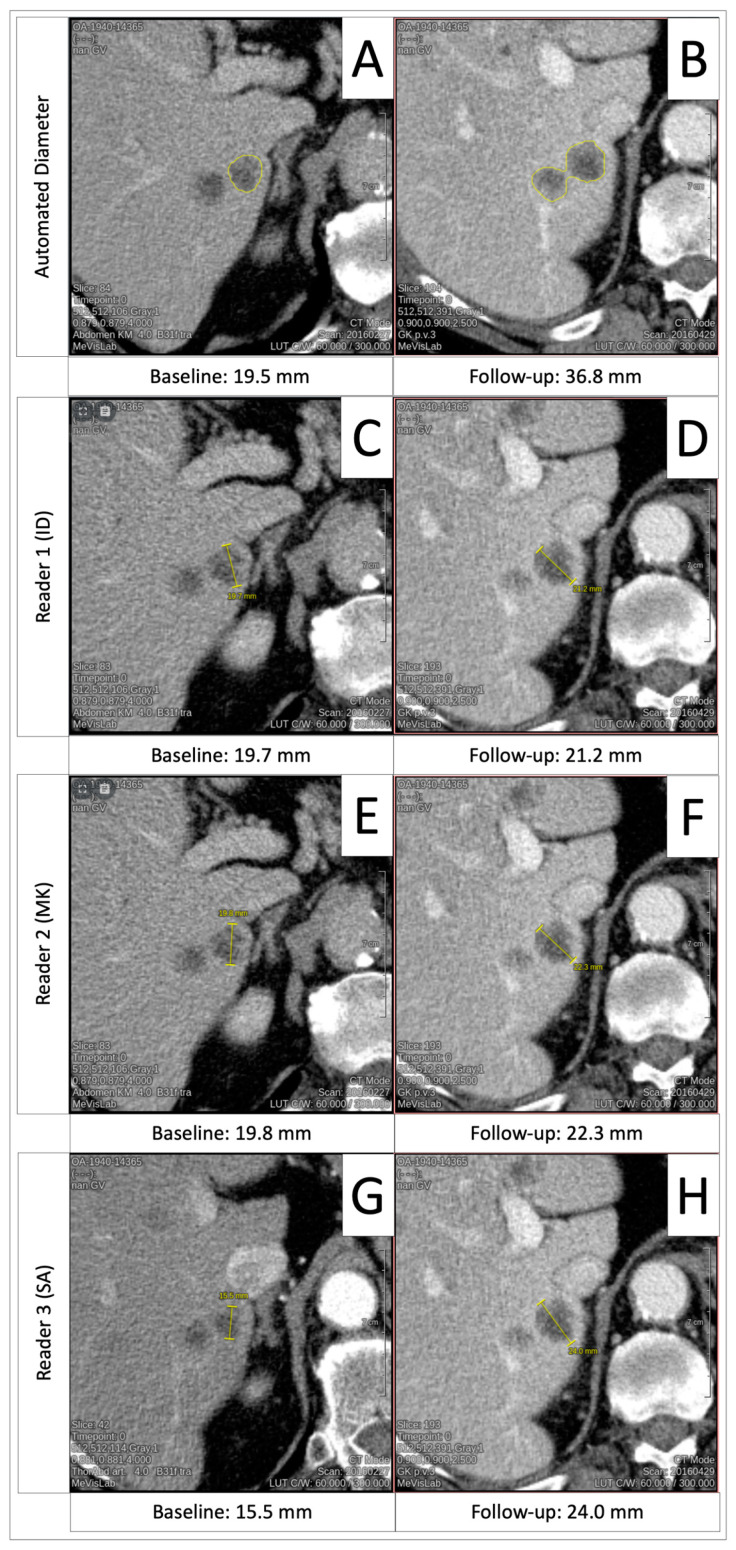
Incorrect automated segmentation. Baseline and follow-up measurements for automated diameter (**A**,**B**), reader 1 (**C**,**D**), reader 2 (**E**,**F**), and reader 3 (**G**,**H**). The algorithm incorrectly outlines the lesion in the follow-up CT (**B**) and includes a metastasis close by, with the resulting artificial growth of the tumor diameter. All three human readers have outlined the correct lesion (**D**,**F**,**H**).

**Figure A4:**
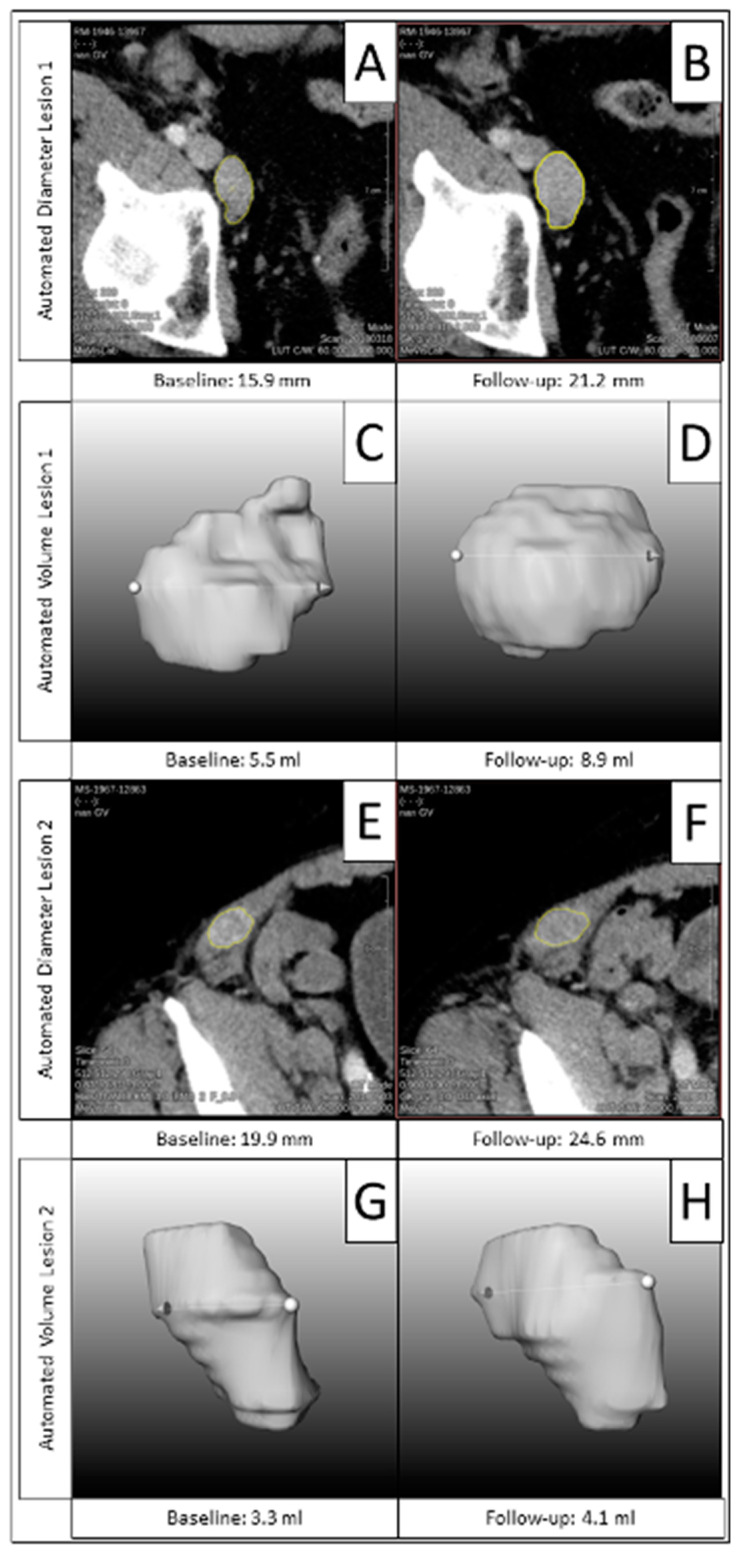
Non-spherical shape of lesion, leading to differences between diameter- and volume-based timepoint response. Baseline and follow-up measurements for automated diameter (lesion 1: (**A**,**B**); lesion 2: (**E**,**F**)) and automated volume (lesion 1: (**C**,**D**); lesion 2: (**G**,**H**)). The timepoint response for the automated diameter indicates progressive disease for both lesions (lesion 1: +33.1%; lesion 2: +23.8%). The timepoint response for the automated volume indicates stable disease for both lesions (lesion 1: +61.4%; lesion 2: +23.4%).

For the patient-level timepoint response, full agreement was observed for 34 patients (59%); minor deviations occurred in 14 patients (24%) across readers (I.D., M.K., S.A.), automated diameters, and automated volumes. In 10 patients (17%), major deviations (differences between non-progressive disease (CR, PR, SD) and progressive disease (PD)) were present. In three of these cases, full agreement existed between the readers (patients 28, 44, 50), but a deviation occurred in the automated diameter (patient 28) or automated volume timepoint responses (patients 28, 44, 50 (see Table 6)). A detailed analysis of these 10 cases identified three primary causes of the deviation. In 3/10 cases, the lesion growth was close to the 20% diameter/73% volume threshold (see Figure A1 in Appendix B). For instance, the diameter/volume changes in subject 17 were +19.7%, +21.5%, +19.9%, +17.0%, and +58.9% for readers A.D., M.K., and S.A. regarding the automated diameter and automated volume, respectively. In 5/10 cases, lesions were missed or mismeasured in the follow-up CT by the readers (1/10 subjects) or by the automated algorithm (4/10 subjects, example illustrated in Figure A3 in Appendix B). In 2/10 cases, non-spherical lesion shapes resulted in differences between the diameter- and volume-based timepoint responses (see Figure A4 in Appendix B).

## 4. Discussion

This study evaluated the reliability of an algorithm for the automated RECIST evaluation of target lesions in follow-up CT, comparing it with manual measurements by multiple radiologists from different institutions, in a sample of 58 patients with metastatic melanoma. Initially, the intra- and inter-reader reliability for manual diameter measurements at baseline and the first follow-up, along with the resulting timepoint response variability, was analyzed. The results demonstrated excellent intra- and inter-reader reliability, contrary to the commonly suggested variability in manual measurements between different readers and institutions [22,23], supporting the RECIST committee’s diameter-based workflow [1]. Diameter-based response evaluation remains the gold standard in many clinical trials, aligning with publications that confirm its reliability [24]. The timepoint responses also showed substantial agreement among the three readers, as indicated by the high Fleiss’ Kappa values, supporting Kuhl et al., who observed high agreement with consistent target lesion selection [25]. However, in 6 of 58 patients, discrepancies in assigning “progressive disease” timepoint responses highlighted potential implications for patient treatment, depending on the study protocol or tumor board decision. These differences are likely due to the threshold-based nature of the RECIST criteria. For instance, a patient with a 19% increase in the lesion diameter is classified as stable (based on the smallest diameter sum at follow-up, with an absolute increase of ≥5 mm), while a 20% increase signifies progressive disease [1,26]. This discrepancy can be especially significant in smaller target lesions, where the full five potential target lesions per RECIST 1.1 cannot be identified, as was the case in our sample. Consequently, the RECIST guidelines suggest that the RECIST criteria alone may sometimes be inadequate in accurately evaluating treatment-induced changes; experienced readers and close clinician collaboration are essential to determine the clinical implications [26].

In the second phase, the automated diameter responses from the proposed algorithm were compared to manual measurements. The results showed excellent agreement between the manual and automated measurements at both the lesion and patient levels, with the ICC values remaining > 0.90. The differences between the mean diameter measurements of each reader and the automated diameters were ≤2 mm at the lesion level and ≤4 mm at the patient level. When calculating the mean diameters across all readers and comparing them to the automated diameters, the differences were ≤ 1 mm at the lesion level and ≤ 2 mm at the patient level. The agreement for the timepoint response was moderate to almost perfect, although, in eight cases, the automated evaluations disagreed with at least one reader regarding response classification. As shown in Figure A3 (Appendix B), the algorithm either missed or mismeasured lesions in the follow-up CT in four of these cases. In the remaining four cases, the discrepancies were due to manual measurement errors or RECIST’s threshold-based nature. A recent EORTC and ESOI joint publication emphasized that automation in RECIST evaluations can reduce variability, yet technical challenges may necessitate human adjudication [26]. Our findings confirm this, showing that automated diameters are generally reliable but that technical limitations may necessitate visual inspection by experienced readers [27,28,29,30].

Third, as automated tracking and segmentation provide not only diameters but also 3D masks for tumor volumes, fully automated volumetric RECIST evaluations were compared to automated diameter evaluations to assess the potential response classification changes. The agreement with manual evaluation was moderate to substantial, and the agreement with automated diameter evaluation was substantial to almost perfect. In 7 of 58 cases, the volumetric RECIST differed from the diameter-based RECIST at the patient level, with three discrepancies in “progressive disease” classification. These differences appeared primarily in large or irregularly shaped lesions, supporting Greenberg et al.‘s conclusion that the diameter-based RECIST may not fully capture changes in non-spherical tumors, whereas volumetric response evaluations may more accurately depict changes in irregularly shaped, large lesions [31]. Additionally, automated volumetric segmentation offers the potential for whole-body tumor burden quantification and radiomics as additional response markers [25,27,32,33].

Our study has limitations. The sample lacked cases with lesions that split or merged during treatment. Future studies could address this by expanding the training dataset. Furthermore, the randomly selected sample did not include all metastatic sites; for example, splenic and cerebral metastases were absent, and osseous metastases were underrepresented. However, we believe that the sample represents the key metastasis types seen in whole-body CT imaging. Cerebral metastases were excluded, as they are typically followed by cerebral CT or MRI rather than whole-body CT. The timepoint response followed RECIST 1.1 rather than iRECIST, as the cohort included both immunotherapy and targeted therapy patients, and thus iRECIST response categories such as “immune unconfirmed progressive disease” were not applicable. Preselecting the target lesions to assess the intra- and inter-reader reliability led to the exclusion of factors influencing variability in the target lesion sum and response evaluations [25], limiting the results’ interpretability. New lesions, another variability factor, were also outside this study’s scope. Future studies may address whole-body lesion segmentation and advanced detection algorithms [25,34], although these challenges remain best handled with radiologist oversight [26,35].

## 5. Conclusions

The automated diameter measurement of preselected target lesions in follow-up CT is reliable and may accelerate RECIST evaluation. However, factors affecting response reproducibility—such as target lesion selection and new lesion interpretation—persist and require further research into whole-body lesion segmentation and advanced detection algorithms. For now, radiologist involvement remains essential.

## Figures and Tables

**Table 1:** Patients’ characteristics.

	n	%
**Gender**		
Female	25	43
Male	33	57
**Age** (years, [SD ^1^])	62.8 [12.9]	
**Treatment**		
Immunotherapy	40	69
Targeted therapy	18	31
**Number of target lesions**	114	
Soft tissue	39	34
Lymph node	28	26
Lung	20	18
Liver	18	16
Adrenal gland	7	6
Osseous	2	2
**Median number of target lesions per patient** (n, [IQR ^2^])	2 [1.75]	
**Mean baseline lesion diameter** (mm, [SD])	27.2 [0.85]	
**Mean follow-up lesion diameter** (mm, [SD])	21.88 [0.43]	

^1^ Standard deviation. ^2^ Interquartile range.

**Table 2:** Mean diameter differences between first and second reading session.

	Mean Diameter Difference (mm)	SD (mm)	*p*
**Lesion level**			
**Reader**			
ID (BL ^1^)	0.62	5.03	0.19
ID (FU ^2^)	0.15	4.06	0.69
MK (BL)	0.11	2.21	0.59
MK (FU)	0.10	6.26	0.86
SA (BL)	0.40	2.53	0.10
SA (FU)	0.56	2.11	**0.01**
**Patient level**			
**Reader**			
ID (BL)	1.23	7.37	0.21
ID (FU)	0.30	5.20	0.66
MK (BL)	0.22	3.07	0.59
MK (FU)	0.21	9.38	0.87
SA (BL)	0.78	3.38	0.08
SA (FU)	1.01	2.48	**<0.01**

^1^ Baseline. ^2^ Follow-up.

**Table 3:** Intra-reader reliability of diameter measurements at lesion and patient level.

	ICC ^1^	95% CI ^2^
**Lesion level**		
**Reader**		
ID (BL)	0.97	0.96–0.98
ID (FU)	0.99	0.99–0.99
MK (BL)	0.99	0.99–1.00
MK (FU)	0.99	0.99–1.00
SA (BL)	0.99	0.99–1.00
SA (FU)	0.99	0.99–1.00
**Patient level**		
**Reader**		
ID (BL)	0.99	0.99–1.00
ID (FU)	0.99	0.99–1.00
MK (BL)	1.00	1.00–1.00
MK (FU)	0.99	0.98–0.99
SA (BL)	1.00	1.00–1.00
SA (FU)	1.00	1.00–1.00

^1^ Intraclass correlation coefficient. ^2^ Confidence interval.

**Table 4:** Inter-reader reliability of diameter measurements and timepoint response at lesion and patient level, exclusive and inclusive of automated diameters.

Diameter Measurements	ICC	95% CI
**Radiologists only**		
Lesion level		
BL	0.99	0.99–1.00
FU	0.99	0.99–1.00
Patient level		
BL	1.00	0.99–1.00
FU	1.00	1.00–1.00
**Inclusive of automated diameters**		
**Reader**		
Lesion level		
BL	0.99	0.99–0.99
FU	0.98	0.97–0.98
Patient level		
BL	0.99	0.99–1.00
FU	0.99	0.99–0.99
**Timepoint response**	**Fleiss’ k** ^3^	**95% CI**
**Radiologists only**		
Lesion level	0.79	0.79–0.79
Patient level	0.68	0.68–0.68
**Inclusive of automated diameters**		
Lesion level	0.66	0.66–0.66
Patient level	0.69	0.69–0.69
**Inclusive of automated volumes**		
Lesion level	0.66	0.66–0.67
Patient level	0.67	0.67–0.68
	**Cohen’s k**	**95% CI**
**All readers mean vs. AD** ^1^		
Lesion level	0.67	0.56–0.78
Patient level	0.76	0.61–0.90
**All readers mean vs. AV** ^2^		
Lesion level	0.69	0.59–0.80
Patient level	0.73	0.58–0.87
**Automated diameters vs. volumes**		
Lesion level	0.81	0.72–0.90
Patient level	0.81	0.67–0.94

^1^ Automated diameters. ^2^ Automated volumes. ^3^ Kappa.

**Table 5:** Mean diameter differences between mean diameters of readers and automated diameters, split by timepoint, at lesion and patient level.

	Mean Diameter Difference (mm)	SD (mm)	*p*
**Lesion level**			
**Reader**			
ID vs. AD (BL)	0.36	6.60	0.56
ID vs. AD (FU)	0.36	10.62	0.72
MK vs. AD (BL)	2.21	8.00	**<0.01**
MK vs. AD (FU)	0.37	10.62	0.71
SA vs. AD (BL)	2.16	8.00	**0.01**
SA vs. AD (FU)	0.88	12.16	0.44
All readers vs. AD (BL)	1.01	7.00	0.13
All readers vs. AD (FU)	0.44	10.91	0.67
**Patient level**			
**Reader**			
ID vs. AD (BL)	0.71	9.90	0.56
ID vs. AD (FU)	0.70	14.30	0.71
MK vs. AD (BL)	4.27	11.05	**0.01**
MK vs. AD (FU)	1.78	16.09	0.40
SA vs. AD (BL)	0.97	11.26	0.51
SA vs. AD (FU)	0.09	15.56	0.97
All readers vs. AD (BL)	1.99	9.92	0.13
All readers vs. AD (FU)	0.86	14.70	0.66

**Table 6:** Mean diameter differences between mean diameters of readers and automated diameters, split by timepoint, at lesion and patient level.

	Timepoint Response						Timepoint Response				
Patient	ID	MK	SA	AD	AV	Patient	ID	MK	SA	AD	AV
1	PR	PR	PR	PR	PR	30	PR	CR	PR	PR	PR
2	PR	PR	PR	PR	PR	31	SD	SD	SD	SD	SD
3	SD	SD	SD	SD	SD	32	PR	PR	PR	PR	PR
4	PR	PR	PR	PR	PR	33	CR	CR	PR	PR	PR
**5**	SD	SD	PD	PD	SD	34	PR	PR	PR	PR	PR
6	PR	PR	PR	PR	PR	**35**	PD	SD	PD	SD	SD
7	PR	PR	PR	PR	PR	36	PR	PR	PR	PR	PR
8	SD	SD	SD	SD	SD	37	SD	SD	PR	SD	SD
9	SD	SD	SD	PR	SD	38	PR	PR	PR	PR	PR
10	SD	SD	SD	SD	SD	39	SD	SD	SD	SD	SD
11	PR	PR	PR	PR	PR	40	CR	CR	PR	PR	PR
12	PR	PR	PR	PR	PR	41	CR	CR	PR	PR	PR
13	SD	SD	SD	SD	SD	42	SD	SD	SD	SD	SD
14	SD	SD	SD	SD	SD	43	PD	PD	PD	PD	PD
15	PR	SD	SD	SD	SD	**44**	PD	PD	PD	PD	SD
16	PR	SD	SD	SD	SD	**45**	SD	SD	PD	SD	SD
**17**	SD	PD	SD	SD	SD	46	SD	SD	PR	PR	PR
18	PD	PD	PD	PD	PD	**47**	SD	SD	PD	PD	SD
19	PR	SD	PR	SD	PR	48	SD	SD	SD	SD	SD
20	SD	SD	SD	SD	SD	49	PR	PR	PR	PR	PR
21	PR	PR	PR	PR	PR	**50**	PD	PD	PD	PD	SD
22	SD	SD	SD	SD	PR	51	PR	PR	PR	PR	PR
**23**	PD	SD	PD	PD	PD	52	CR	CR	PR	PR	PR
24	PD	PD	PD	PD	PD	53	PR	PR	PR	PR	PR
25	SD	SD	SD	SD	SD	**54**	PD	PR	PR	PD	PD
26	PR	CR	PR	PR	PR	55	PR	CR	PR	PR	PR
27	PD	PD	PD	PD	PD	56	PR	PR	PR	PR	PR
**28**	PD	PD	PD	PR	PR	57	PD	PD	PD	PD	PD
29	PR	PR	PR	PR	PR	58	PD	PD	PD	PD	PD

CR/dark green background: complete response. PR/light green background: partial response. SD/yellow background: stable disease. PD/red background: progressive disease.

**Figure A2:**
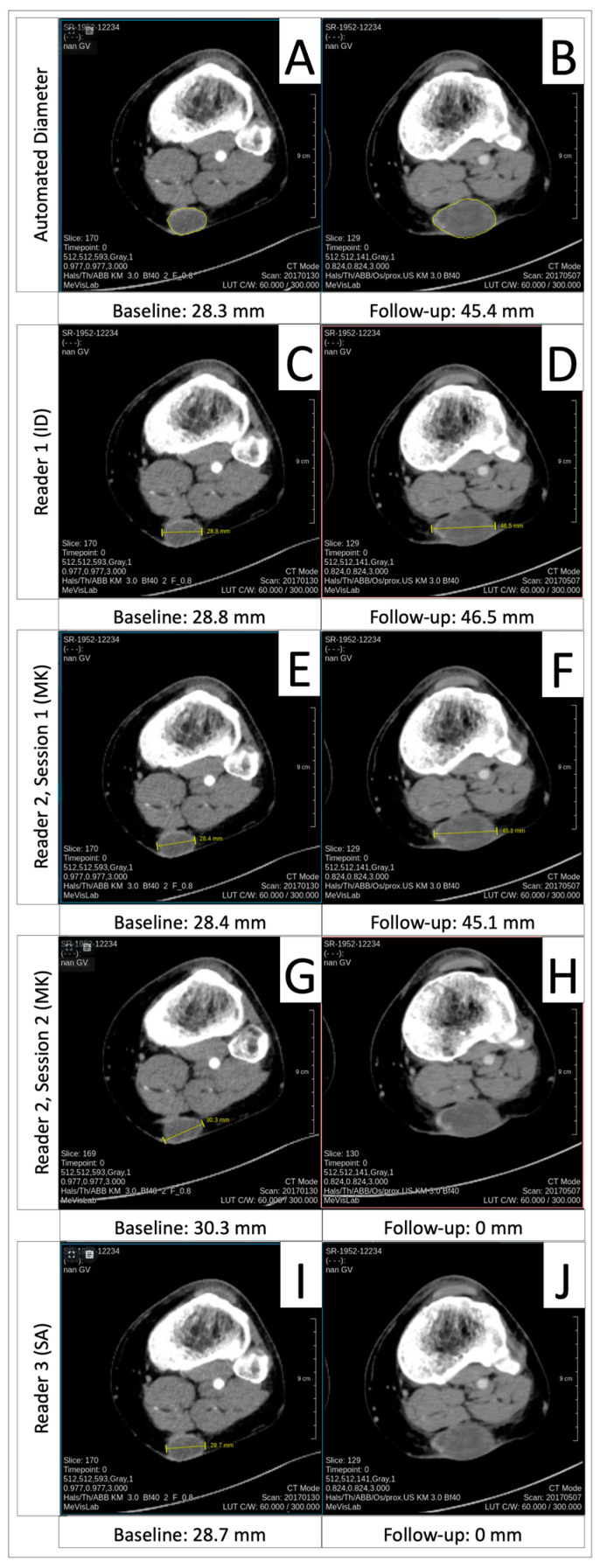
Examples for intra- and inter-reader variability. Baseline and follow-up measurements for automated diameter (**A**,**B**), reader 1 (**C**,**D**), reader 2 session 1 (**E**,**F**), reader 2 session 2 (**G**,**H**), and reader 3 (**I**,**J**). Baseline measurements (**A**,**C**,**E**,**G**,**I**) are very close, with low inter- and intra-reader variability. However, follow-up measurements show high inter-reader variability ((**B**,**D**), vs. (**H**,**J**)) and intra-reader variability (**F**,**H**).

## Data Availability

The data may be made available after a reasonable and well-justified request to Felix Peisen. The data cannot, however, be made freely available to the public, due to privacy regulations. Codes and materials used in this study may be made available for the purposes of reproducing or extending the analysis, pending materials transfer agreements.

## Abbreviations

AD	Automated diameter
AV	Automated volume
BL	Baseline
CI	Confidence interval
CNN	Convolutional neural network
CR	Complete response
CT	Computed tomography
EORTC	European Organisation of Research and Treatment in Cancer
ESOI	European Society of Oncologic Imaging
FU	Follow-up
ICC	Intraclass correlation coefficient
IQR	Interquartile range
k	Kappa
mm	Millimeter
MRI	Magnetic resonance imaging
n	Number
nnUNet	“No-New-Net”
NTL	Non-target lesion
PD	Progressive disease
PR	Partial response
RECIST	Response Evaluation Criteria in Solid Tumors
SD	Stable disease
SD	Standard deviation
TL	Target lesion
TPR	Timepoint response

## Appendix A. Scan Parameters and CT Scanner/Vendor Details

In-house staging CT was performed on four CT scanners (Sensation 64, SOMATOM Definition AS, SOMATOM Definition Flash, SOMATOM Force, Siemens Healthineers, Erlangen, Germany) and one PET-CT scanner (Biograph128, Siemens Healthineers, Erlangen, Germany). The in-house whole-body staging protocol was used with a scan field from the skull base to the middle of the femur, with patients laid in a supine position, arms raised above the head. Scanning was performed during the portal venous phase after the administration of a body weight-adapted contrast medium through the cubital vein. Attenuation-based tube current modulation (CARE Dose, reference mAs 240) and tube voltage (120 kV) were applied. The following scan parameters were used: SOMATOM Force—collimation 128 × 0.6 mm, rotation time 0.5 s, pitch 0.6; Sensation 64—collimation 64 × 0.6 mm, rotation time 0.5 s, pitch 0.6; SOMATOM Definition Flash—collimation 128 × 0.6 mm, rotation time 0.5 s, pitch 1.0; SOMATOM Definition AS—collimation 64 × 0.6 mm, rotation time 0.5 s, pitch 0.6; Biograph128—collimation 128 × 0.6 mm, rotation time 0.5 s, pitch 0.8. The slice thickness as well as increment were set to 3 mm. A medium smooth kernel was used for image reconstruction. Seventeen external baseline CT scans were also included to obtain a more realistic sample and reduce sample bias. Detailed information about the contrast medium phase, tube current, and tube voltage is not available for these cases. For details about the distribution of the CT scanners and vendors, see Table A1.

**Table A1:** Distribution of CT scanners.

			Number of Patients		
Cohort	Scanner	Vendor	Baseline	Follow-Up	Total
In-house	SOMATOM Definition AS+	Siemens	6	10	16
	SOMATOM Definition Flash	Siemens	3	1	4
	SOMATOM Force	Siemens	22	23	45
	Sensation 64	Siemens	5	7	12
	Biograph128	Siemens	11	11	22
External	Aquillion One	Canon	1	1	2
	Lightspeed VCT	GE	1		1
	Optima CT540	GE	1		1
	Ingenuity Core	Philips		1	1
	Biograph64	Siemens	1		1
	Emotion 16	Siemens	2	1	3
	Scope	Siemens		1	1
	SOMATOM Definition AS	Siemens	4	1	5
	SOMATOM Definition Edge	Siemens	1		1
	SOMATOM Definition Flash	Siemens		1	1
Total			58	58	116
